# Millet vs rice: an evaluation of the farming/language dispersal hypothesis in the Korean context

**DOI:** 10.1017/ehs.2020.13

**Published:** 2020-05-05

**Authors:** Jangsuk Kim, Jinho Park

**Affiliations:** 1 Department of Archaeology and Art History, Seoul National University, Seoul, South Korea; 2 Department of Korean Language and Literature, Seoul National University, Seoul, South Korea

**Keywords:** Farming/language dispersal hypothesis, diffusion, migration, millet, rice

## Abstract

The ‘farming/language dispersal hypothesis’ was originally developed to explain the spread of the Neolithic economy and material culture into Europe. Recently, this hypothesis has been applied towards explaining the dispersal and divergence of East Asian languages. However, interpretations depend on what prehistoric cultivar is chosen by linguists as having been related with the spread of language. In understanding the appearance of the proto-Koreanic and proto-Japonic languages in Korea, millet and rice, which appeared in Korea around 3500 and 1300 BCE, respectively, have been emphasized by linguists. We assess these linguistic arguments. We first review how European archaeologists have understood the spread of farming into Europe, where the farming/language dispersal hypothesis was originally developed, and how archaeology has wrestled with the issues of diffusion and migration. Then we move on to evaluating linguistic hypotheses about the dispersal and split of proto-Koreanic and proto-Japonic. Our evaluation of the ‘millet hypothesis’ and the ‘rice hypothesis’ suggests that rice is a more plausible candidate for explaining the dispersal of proto-Koreanic to Korea. Meanwhile, viewing the introduction of slender daggers to Korea as another dispersal of language to Korea needs more scrutiny.

## Graphical abstract

**Figure:**
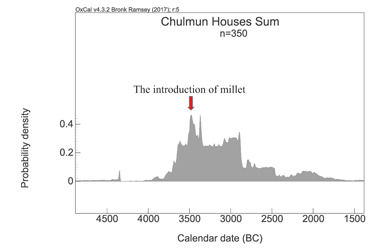

**Media summary:** Archaeological records suggest that the spread of millet to Korea around 3500 BCE had little to do with language dispersal.

In explaining dispersal and divergence of languages in the past, historical linguistics recently has paid special attention to the spread of farming. This tendency largely arises from an interest in Renfrew and Bellwood's archaeological works on the spread of farming to Europe at the beginning of the Neolithic (e.g. Bellwood 2001, 2005; Renfrew 2003). Renfrew and Bellwood argue that the spread of farming into Europe was led by the westward migration of Neolithic farmers from Southwest Asia and that the dispersal of farmers resulted in the spread of the proto-Indo-European language. This hypothesis is called the ‘farming/language dispersal hypothesis’ (hereafter FLDH) (Bellwood 2005). The FLDH has had a significant impact on historical linguistics, which relies on prehistoric archaeology to understand the formation, spread and divergence of languages.

Some linguists have attempted to apply this model to other regions, including East Asia (e.g. Robbeets 2017a, d). In the East Asian context, the major focus has been on rice, which originated in South China (specifically, the Yangtze River Basin) and millet from North China. In particular, the spread and split of proto-Koreanic and proto-Japonic are thought to have been critically related to the spread of these cultivars. However, the timing and processes of the dispersals and divergence of these languages have been a point of contention among linguists. Whitman (2011) suggests that the Japonic language family was first brought by rice farmers to the Korean Peninsula around 1500 BCE and then to Japan after 950 BCE, as farmers in Korea moved to Japan. He also suggests that the arrival of Koreanic in Korea was associated with the spread of the Korean-style bronze dagger culture from present-day northeast China to Korea around 300 BCE. In contrast, Robbeets (2017d) argues that proto-Koreanic speakers entered the Korean Peninsula as millet was introduced from Liaoning, China to the peninsula around 3500 BCE and that the Koreanic and Japonic languages initially split during this time. She also suggests that approximately two millennia later, when rice was introduced to Korea around 1300 BCE, rice farmers from Shandong and Liaodong, China brought the Japonic language to the peninsula. Around 800 BCE, she argues, rice farmers in Korea moved to the Japanese Archipelago and brought with them the Japonic language.

The contention between Whitman and Robbeets appears to arise not only from different linguistic views of the proto-Koreanic and proto-Japonic languages, but also from how they apply archaeological research in their studies. In this paper, we discuss why the two views differ in understanding the dispersal and split of the Koeranic and Japonic languages. We begin by reviewing archaeological studies on migration and diffusion, two key concepts for understanding the nature of the spread of farming. Then, we discuss the archaeology of Korea to assess the two contrasting linguistic arguments.

## Archaeological understandings of migration, diffusion and the spread of farming

### Debate on the spread of farming to Europe

The FLDH is now at the centre of a heated controversy in European archaeology (Kristiansen 2005) and linguistics (Chang *et al.* 2015; Hammarström 2010; Ross 2006). To understand the nature and logic of the FLDH, we here briefly review how European archaeology has viewed the Neolithization of Europe. While European archaeologists have long been interested in the Neolithization of Europe since the time of V.G. Childe, it was not until the 1970s and 1980s that archaeologists began to focus their attention on *how* farming spread throughout Europe – that is, the mechanisms behind the spread of farming. The spread of farming in Europe can roughly be divided into three perspectives: migrationist, adoptionist and interactionist.

Migrationist views have the longest history. Ammerman and Cavalli-Sforza (1973, 1984) have suggested that Neolithic farmers spread through a series of colonizations of neighbouring lands, characterizing the spread of agriculture as a ‘wave of advance’. Using radiocarbon dates, they suggested that Neolithic adaptations and material culture spread gradually across Europe and argued that this dispersion took the form of human colonization. They also suggested that this pattern was best explained by rates of population growth and that individual episodes of migrations resembled ‘waves’ of population expansion. This model has had a critical impact on thinking about the spread of not only human populations but also agriculture. However, this model has also been criticized for adhering to Childe's simplistic notion of European Neolithization (Price 2000; Price *et al*. 1995; Whittle 1996). This model essentially describes how farmers spread across Europe, paying little attention to regional diversity, changes in material culture and causal mechanisms.

The notion of the farmer as colonizer has continued to influence later studies examining the spread of farming. Several refined versions of the colonization model that concentrate to a greater degree on the rapidity of the spread of farming have been put forward by researchers. These models often suggest that long-distance migration or ‘leapfrog colonization’, which entailed leaping strides over long distances and ‘frontiers’ rather than a gradual expansion of populations into neighbouring lands, was the characteristic mechanism of the Neolithic spread (Bogucki 1988, 2000; Dennell 1985; Keeley 1992; Milisauskas and Kruk 1989; Moore 1985). These models aim to explain the apparent rapidity of the spread of farming. Nevertheless, since they focus solely on the existence of ‘frontiers’, these models rarely attempt to explain what happened to Mesolithic foragers when farmers migrated into their lands. Indigenous foragers are implicitly seen as having been expelled to marginal areas, leaving uninhabited areas open to occupation by farmers (for exceptions, see Bogucki 1988; Moore 1985).

Adoptionists, focusing on the presence of indigenous hunter–gatherers in Europe rather than frontiers, suggest that the spread of agriculture resulted from indigenous adoption by Mesolithic populations. Central to this perspective is the active role indigenous hunter–gatherers played in the spread of farming, a notion that migrationists have largely neglected. For example, Whittle (1996) suggests active adoption by indigenous Mesolithic hunter–gatherers of Neolithic ideas, animals and plants of Middle Eastern origin.

Meanwhile, interactionists take an eclectic view. Assuming both the existence of agricultural frontiers and an active role for indigenous Mesolithic hunter–gatherers, they focus on interactions between indigenous foragers and migrant farmers. Gregg (1988, 1991) argues that interactions between farmers and foragers produced benefits for each group. In her terminology, ‘indirect food production’ played an important role in the process of the spread of farming. Zvelebil and Rowley-Conwy (1984) argued that various exchanges took place between foragers and farmers before foragers adopted agriculture. Zvelebil and Lillie (2000), synthesizing existing models of forager–farmer interaction, attempt to explain various interactions between indigenous foragers and migrant farmers in what they call the ‘forager–farmer frontier zone’. Although the history of these models is brief, the forager–farmer interaction models provide a new perspective on the study of the spread of agriculture by focusing on the dynamic reactions of foragers to farming.

Both the indigenous adoption and interaction models emphasize the role of indigenous foragers in the spread of farming in Europe, a factor that migrationists hardly address. By the early 2000s, many European archaeologists seemed to lean towards accepting the existence (and significance) of forager–farmer interaction and appeared to have reached a consensus on two points. First, a single mechanism cannot explain every transition from foraging to farming across the vast area of Europe, but instead diverse mechanisms were involved in the processes of transition. Second, the simple dichotomy between colonization and indigenous adoption is problematic. Both colonization by farmers and the reactions of indigenous foragers varied in different places and some kind of ‘contact’ between the two existed throughout the process of the spread of farming.

It was under these circumstances that the FLDH broke into European archaeology in the 1990s and early 2000s (e.g. Bellwood 2001). The FLDH was highly controversial because it not only takes an extreme form of the migrationist argument but also connects the spread of farming directly to the dispersal of the proto-Indo-European language, a topic that had rarely been discussed in archaeology. It was first proposed by Renfrew (1987) in the late 1980s, but was not very influential in European archaeology until genetic studies that supported this hypothesis were published in the early 2000s (e.g. Forster and Toth 2003). While the FLDH was originally Europe-specific, Bellwood (2005) soon attempted to apply it to Africa, Asia and America, arguing that farming and related cultivars were mostly spread through processes of migration. Not surprisingly, many archaeologists immediately pointed out many of the epistemological and empirical problems with this argument (e.g. Kristiansen 2005). In contrast, some historical linguists seemed to welcome and adopt it as a theoretical basis for inferring the timing and routes of language dispersal in many areas.

### General considerations of migration, diffusion and adoption

Simply put, one of the central points of the debate between migrationist (including the FLDH), adoptionist and interactionist perspectives is the question of how non-local elements appear in a particular area. When non-local elements such as ideas, technologies, styles and items appear in an area, four heuristic mechanisms can be taken into consideration, each of which has a different implication: either the element was (a) brought by migrants, (b) moved through some sort of exchange, (c) copied or adopted by local people, or (d) made by migrants and/or local people who learned how to make it through intensive interaction (Hegmon *et al*. 2000: 218). In archaeology, it is important to distinguish these different mechanisms because the archaeological picture and our understanding of the past are critically affected by the way in which a model uses these mechanisms, as we have seen in the debate between the migrationists and adoptionists. Focusing on diffusion and migration, we here discuss the different processes implied by these two mechanisms and how they can be archaeologically distinguished.

#### Diffusion and adoption

Although diffusion is currently not a popular concept applied towards explaining culture change, many archaeologists still work with it, interpreting it differently and with different theoretical concerns. Information flow, acculturation, cultural transmission and assimilation are alternative but related concepts that are often used in place of diffusion. Despite some slight differences in nuance, all of these concepts focus on the strategic behaviour of senders and recipients (e.g. Aldenderfer 1993; Hegmon 1998; Kim 2001; Spencer 1993). A common assumption is that people ‘accept’ or ‘adopt’ other people's technology, information, style, etc. either consciously or unconsciously, during periods of interaction or contact. In other words, a prerequisite of the diffusion of new technologies, ideas or styles in contrast to migration is a decision made by recipients to accept them. Even when potential recipients are cognizant of the styles or technologies of neighbours, these are not diffused unless the recipients adopt them (Kim 2001, 2003a).

These decisions depend on various economic and sociopolitical factors, and motivations for the adoption of new cultural elements or technologies also vary. For example, when a hunter–gatherer group faces the problem of decreasing productivity or experiences a population–resource imbalance, it may attempt to adopt farming as a solution. Even when there is no problem with the existing subsistence system, hunter–gatherers may adopt farming as a supplementary technology in order to diversify their food resources. As Hayden (1995) and Clark and Blake (1994) suggest, they may also adopt crop cultivation to produce new prestige/ritual items. In any of these scenarios, there is no reason to assume that linguistic changes must accompany the spread (or spatial expansion) of farming.

#### Migration

Non-local technology and culture may also be introduced via the migration of people. Anthropological, sociological and demographic studies of migration have shown that most migrations of sedentary groups occur when migrants can no longer remain where they were living previously (Kershaw 1978; Kim 2002b). In this sense, migration does not necessarily refer to conquest or invasion, but may be the only strategy that people who cannot stay in their homelands can choose, particularly when the cost of staying in their homeland exceeds the cost of leaving it (Kim 2002b). Unless the migrants settle uninhabited areas, it is not surprising that migrations often have little, if any, impact on the existing material culture of host societies that is archaeologically visible on a prehistoric timescale. Demography and economic geography pay close attention to the relationships between migrants and hosts, especially how migrants, usually minorities, adjust to or conflict with host societies. Regardless of what eventually happens afterwards, migration will predicate interaction between migrants and indigenous populations (Kim 2002b), including incorporation or acculturation, power struggles and/or avoidance. Power relations between migrants and hosts affect whether or not the migrants maintain or modify their way of life and in what ways; they also affect the degree and nature of the impact that migrant cultures exert on host societies. Therefore, without understanding the actions and reactions of the two groups and the power relations between them, the study of migration can provide only a limited explanation of culture change. Migration is not, in this sense, a one-dimensional phenomenon or event, but a multi-faceted process, and thus, should not be confused with conquest or invasion (Kim 2002b).

Depending on power relations (not only sociopolitical but also economic and cultural) between migrants and indigenes, the processes and consequences of migration vary significantly. One of the major factors affecting this relation is the migrant–indigene ratio in terms of population size, although it is not population size per se but socioeconomic–cultural power relations that may arise as a result of disparities in population size that may determine the processes and final consequences of migration (Kim 2002b).

In one scenario, the size of the migrant group might be too small to have a considerable influence on the indigenous society. The former is then integrated into the latter and must change its (i.e. the migrants’) material culture and way of life. If this process ends rapidly, it may be almost impossible to document migration from archaeological data (Hegmon *et al*. 2000; Cordell 1995; Koenig and Diarra 2000). Secondly, the migrants might enter part of a territory occupied by an indigenous population with loose boundaries, and some blending of the two cultures might occur between the two sub-areas. A third possibility is that the migrants’ culture dominates the area. This occurs when the migrants’ population size (or power) is great enough to replace the indigenous culture. It should be noted that this is not synonymous with ethnic replacement but suggests that indigenes assimilate or accept migrants’ culture either actively or through force. A fourth scenario could be that the migrants’ culture is widespread with no clear boundary in the first place but nonetheless impacts indigenous traditions, thus altering the culture of the whole area. Fifth, both migrants and the indigenous population may occupy discrete areas and a boundary between them becomes fixed, dividing an area into two social units. A sixth possibility is that the migrants cannot cross the boundaries of the indigenous group, and thus, can only occupy marginal areas. The host population may be resistant to accepting new residents, denying them access to necessary resources. Finally, in contrast, the migrants might be so powerful that they overtake the central territory occupied by the indigenous population, expelling the indigenes to marginal areas. From these examples, it can be suggested that the relationship between migrants and indigenes is one of the most important factors in determining how the two groups interact and in large part determines the consequences of migration and its archaeological outcomes (Kim 2002b).

#### Archaeologically distinguishing the two

Despite the necessity of distinguishing diffusion and migration, one difficulty in the archaeological study of migration/diffusion is that distinguishing migration from cultural diffusion, information flow, assimilation, interaction and indigenous adoption is an extremely difficult task (Clark 1994; Hegmon *et al*. 2000). Ancient DNA and isotopes analyses of human bones from archaeological sites may be methods that can distinguish migration from other mechanisms. However, these analyses do not always guarantee a clear conclusion, in that migrant groups cannot always be readily distinguished based on genetic variation or differences in ratios of isotopes.

Despite the limitations of archaeological data, archaeologists have long attempted to tackle this issue. Some archaeologists have suggested some criteria and/or expectations that would aid in identifying migration. Haury (1958) has enumerated three expectations of migration: (a) there is a sudden appearance of a constellation of non-local traits in an area where there had been a continuum; (b) the products of the migrant group reflect both elements borrowed from the hosts and unmistakable elements of their own style (e.g. ceramic design); and (c) an area of possible destination shows a constellation of traits as the normal pattern, and there is a rough chronological correlation between the home and recipient area sites.

Rouse (1958: 64) has also suggested several criteria to evaluate whether population movement is a better explanation for change in the archaeological record than *in situ* development or stimulus diffusion: (a) identify the people that migrate as an intrusive unit; (b) trace this unit back to its homeland; (c) determine that all occurrences of the unit are contemporaneous within the limits of dating techniques; (d) establish the existence of favourable conditions for migrants; and (e) demonstrate that some other hypothesis, such as the independent invention or diffusion of traits, does not more accurately fit the facts. Sanger (1975) added another criterion: (f) establish that all cultural subsystems, rather than a single subsystem, are involved in the cultural change as a package.

Zvelebil (1981) has proposed ‘a number of phenomena [that], when occurring together, probably indicate a migration of a new group of people’ (p. 15). These include: (a) a simultaneous occurrence of a number of attributes in different contexts of material culture that are exogenous to the region and which can be related to another culture group or culture area; (b) initial disappearance or reduction of the previously existing indigenous traits; (c) shift in the location of settlements or in the entire settlement pattern; (d) where applicable, change in the genetic make-up of the population; and (e) the existence of a clear boundary within which such a combination of new elements will be enclosed and beyond which only isolated elements will extend. All of these criteria were suggested long ago, but where other methods are unavailable or difficult to apply, they are still valid and can be applied to detecting migration archaeologically.

## Some issues with the FLDH

While historical linguistics is interested in the timing and routes of language dispersal, it cannot directly address the temporality of these phenomena. Thus, historical linguistics has tended to rely on prehistoric archaeology to provide information on prehistoric migrations. In this sense, from a linguistic perspective, the FLDH, which directly connects linguistic dispersal to the spread of farming, is a very attractive hypothesis and has been widely applied to many areas of the world despite its short history. However, there are some issues to consider.

### Is FLDH a general theory or model?

The FLDH is a hypothesis originally designed to explain the spread of farming and the proto-Indo European Language family in Europe. However, its utility as a general model that can be applied to all parts of the world has yet to be demonstrated. It can be inferred that many occurrences of the spread of farming probably resulted from migrations of farmers, because, as Bellwood (2005) argues, continuous farming at one locale in general tends to result in population increase and also a decrease in soil productivity, providing a motivation for dispersal. However, it is not always the case that the spread of farming and the appearance of non-local cultivars can be explained by the migration of farmers. As seen above, new technology and cultural elements spread for various reasons, and the mechanisms involved in their spread also vary (Kim 2001, 2003). The relationship between the spread of farming and the migration of farmers cannot always be assumed, and therefore, the unconditional application of the FLDH to all regions of the world is not only theoretically but also empirically risky. In fact, Renfrew (2003), the original developer of the FLDH, admits that this hypothesis is not to be applied globally, and Bellwood (2005), who attempted to apply the FLDH to other areas, also states that, although farming is a major factor that may encourage population dispersal, it is not the only one. Even in its application to the European Neolithic, the FLDH is controversial (Chang *et al*. 2015; Kristiansen 2005), and most European archaeologists agree that the migration of farmers cannot comprehensively explain the regional and temporal variability observed in the spread of farming to Europe (Price 2000; Robb 2013). While there are some areas where the migration of farmers accounts for the Neolithization of Europe, there also are many areas where indigenous adoption, assimilation or continuous interaction between farmers and Mesolithic foragers better explains the transition to farming (Robb 2013).

### Was the migration of farmers the only form of migration that led to language dispersal?

A reasonable explanation for the spread of a language family is that its speakers expanded spatially along with it. However, the migration of farmers is not the only form of migration that can be linked to language dispersal. Migrations have taken place throughout human history for various reasons, often unrelated to farming, and on various scales. Population reorganization resulting from factors unrelated to farming may provide a better explanation for language dispersal than farming in some instances. Kristiansen (2005) points out that even though the spread of farming in Europe may have been related with the spread of farmers, as the FLDH assumes, this cannot be the only episode of migration that resulted in the dispersal of proto-Indo-European languages. He emphasizes the importance of the establishment of new international networks connecting Bronze Age societies throughout Eurasia and the Aegean during the early second millennium BC in accounting for the widespread appearance of Proto-Indo-European institutions and material culture. A statistical phylogenetic analysis carried out by Chang *et al*. (2015) suggests that Indo-European language dispersal was not driven by the spread of farming, but rather, that Indo-European languages originated in the Pontic–Caspian steppe and spread together with cultural innovations associated with pastoralism beginning c. 4500–3500 BCE (p. 194). While determining which argument is a better explanation of the spread of the Proto-Indo-European languages is a matter that needs further scrutiny beyond the scope of this paper, in our view, assuming that the timing of a specific language is directly linked to the migration of a farming group, as the FLDH does, is risky, and drawing such conclusions should require strong empirical support.

### Does the migration of farmers automatically lead to linguistic change?

In connecting the spread of farming to language dispersal, the first thing to assess is (a) whether or not the migration of farmers really led to the spread of farming in the given context and (b) whether migration provides a better explanation compared with other mechanisms such as indigenous adoption, cultural diffusion and information flow. Even in the case that migration is the best explanation, we need to also evaluate (c) whether the migration actually led to a linguistic change. In some cases, the spread of farmers may not necessarily have led to an alteration of language in host areas, especially when the size of the migrant group was not large enough and thus integrated into the host society within a short timespan. As discussed above, migration often occurs when people cannot stay in their homelands and leaving is less costly than staying. Therefore, migration involves decisions regarding whether to stay or leave and the decision-making units are usually households (or a community, at the largest scale) rather than regional populations (Kim 2002b). Furthermore, following their arrival in a host's territory, they are often a minority population. Even when the size of migrant groups becomes large owing to a series of migrations, they may not be able to change the culture and language of their host societies. Also, host populations may adopt farming from migrant farmers without changing their language, even in cases in which they may adopt or borrow specific terms associated with farming.

## The spread of millet and rice to the Korean Peninsula

We now return to the linguistic application of the FLDH to explain the movement and split of the proto-Koreanic and the proto-Japonic languages in prehistory. As seen above, at issue are (a) whether millet and rice were dispersed to Korea as a result of migrations of farmers and (b) whether the scale of migration was large enough to lead to dispersals of the proto-Koreanic and Japonic languages.

### The introduction of millet to Korea

In Korea, millet first appeared in the Chulmun Period (6000–1300 BCE). The Chulmun Period was characterized by a hunter–gatherer–fisher economy in which pottery was used, although there was extreme regional diversity in subsistence strategies, stone tool assemblages, pottery styles and the major resources utilized (Ahn *et al*. 2015; Lim 2012; Kim 2003a, 2006). Freshwater fishing and the collection of various plant resources were a major part of the Chulmun subsistence economy, evidenced by findings of stone tools such as net sinkers, fishhooks and points, and the remains of wild plants such as acorns and nuts. The exploitation of sea resources was another important part of the subsistence economy. Shell middens dated from early to the latest Chulmun are densely distributed along the coast and on offshore islands of the Korean Peninsula (Kim 2006, 2010). Considering these various data, many Korean archaeologists have reached a consensus that the Chulmun subsistence economy was a broad-spectrum foraging economy (Ahn 1994; Ahn *et al*. 2015; Kim 2010).

Around 3500 BCE, reliable evidence for millet cultivation appears. Domesticated foxtail and broomcorn millets have been identified from flotation samples (Crawford and Lee 2003; Lee 2011) and impressions on pottery (Nakayama 2014). So far, millets have been reported from 87 features (mostly houses, outdoor features and middens) from 18 sites (see Ahn *et al.* 2015 for details). Since there is no evidence for *in situ* domestication of millet in Korea, it is thought that millet was introduced from northeast China where millet cultivation had been already in practice (Ahn *et al.* 2015).

However, the introduction of millet does not appear to have had a major impact on Chulmun material culture and its subsistence economy. While pottery styles clearly differ between northeast China and the Korean Peninsula, an influx of northeast Chinese pottery styles into Korea has not been detected, and the styles of the two areas remain distinct long after the appearance of millet with little change in Chulmun pottery styles over time (Lim 2012). The stone tool assemblages of Chulmun sites remain unchanged, showing continuing heavy dependence on hunting and fishing and the gathering of wild plants (Ahn *et al.* 2015; Kim 2010). There is also no indication of significant changes in settlement patterns and land-use strategies, and the hundreds of shell middens discovered along the coast and on islands continue to be used throughout the period (Kim 2010). Isotopes analyses on human bones that postdate the introduction of millets also clearly indicate that Chulmun people depended heavily on sea resources and wild plants (An 2006; Bae *et al*. 2013;Choy and Richards 2010). Even setting aside the issue of whether or not the presence of millets in archaeological features clearly indicates that millets were both cultivated and consumed, these lines of evidence suggest that millet cultivation during the Chulmun Period was not a major subsistence strategy but only an auxiliary activity that was added to the existing hunter–gatherer–fisher economy, possibly for the purpose of diversifying resources (Ahn *et al.* 2015; An 2006; Kim 2010).

**Figure 1.**
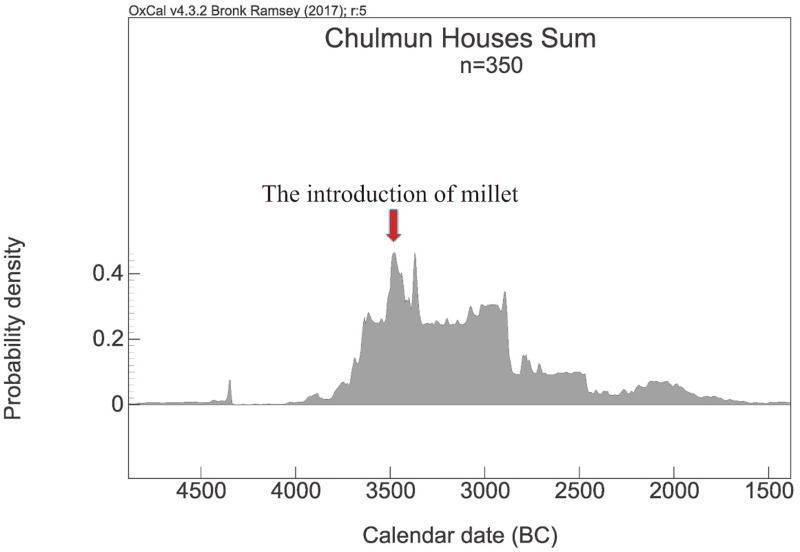
A summed probability distribution of radiocarbon dates from Chulmun houses (*n* = 350). Dates were calibrated and summed using IntCal 13 and OxCal v.4.3.2 (Bronk Ramsey 2017).

After the introduction of millets, both the size of settlements and the number of houses decrease, as shown in the summed probability distributions of radiocarbon dates from houses, and this declining trend continues towards the end of the Chulmun Period (Figure 1). Some researchers have interpreted this change as reflecting population decline or an increase in the mobility of Chulmun hunter–gatherers, which would have probably resulted in lower visibility of archaeological sites (Ahn *et al*. 2015; Lim 2012). Although the cause of this change is not yet clear, it strongly suggests that the introduction of millet around 3500 BCE did not lead to a transition to a farming economy.

### The introduction of rice to Korea and Japan

Around 1300 BC, Korea witnessed dramatic, abrupt changes in both subsistence economies and material culture at a regional level: Chulmun material culture and subsistence economies suddenly disappeared, and the Mumun Period (1300–400 BCE) began. The shapes of dwellings and pottery styles changed fundamentally. New stone tools with new forms and functions appear and dominate the stone-tool assemblage. These marked changes in material culture were accompanied by an abrupt change in the subsistence economy (Kim 2002a, 2003a). The Mumun economy was heavily dependent on rice cultivation, which was new to the region. Not only does rice suddenly dominate archaeobotanical assemblages, but dry rice fields have also been located in the vicinity of many settlement sites (Ahn 2010). In some locales where natural marshes and bogs developed, wet farming was also practised, but dependence on wet farming was still limited compared with dry farming (Ahn 2010; Kim 2003a).

It is widely accepted that Mumun material culture, rice-farming and associated stone-tool technologies moved from northeast China through northern Korea to central and southern Korea (Ahn 2010; Kim 2003a). One striking feature of this shift is that its spread throughout the Korean Peninsula was strikingly rapid, leading to an abrupt regional-scale transition. The subsistence change between the Chulmun and Mumun periods was not the simple addition of rice cultivation to the Chulmun subsistence repertoire. Instead, it was the cessation of a foraging economy coincident with the appearance of rice cultivation, accompanied by marked changes in material culture and settlement patterns (Kim 2003a). Furthermore, there seems to be no continuity in the location of sites between the Chulmun and Mumun periods.

In addition to the rapidity and abruptness of the transition, two important characteristics of the Mumun culture are (a) its spread as a package of material culture and subsistence economy and (b) homogeneity in both the subsistence economy and material culture after the spread throughout the region (Kim 2002a, 2003a). Mumun cultural elements did not spread variably but instead as a package. Although there are some variations in ceramic styles according to the decorations observed on the rims of vessels, the overall shape of the vessels, the structure of ceramic/stone tool assemblages and dwelling shapes are strikingly homogeneous throughout central and southern Korea (Kim, 2002a). The regional diversity in stone tool assemblages and subsistence strategies during the Chulmun Period also disappears: the subsistence economy was now firmly concentrated on rice farming and stone tool assemblages become homogenous throughout the region.

**Figure 2.**
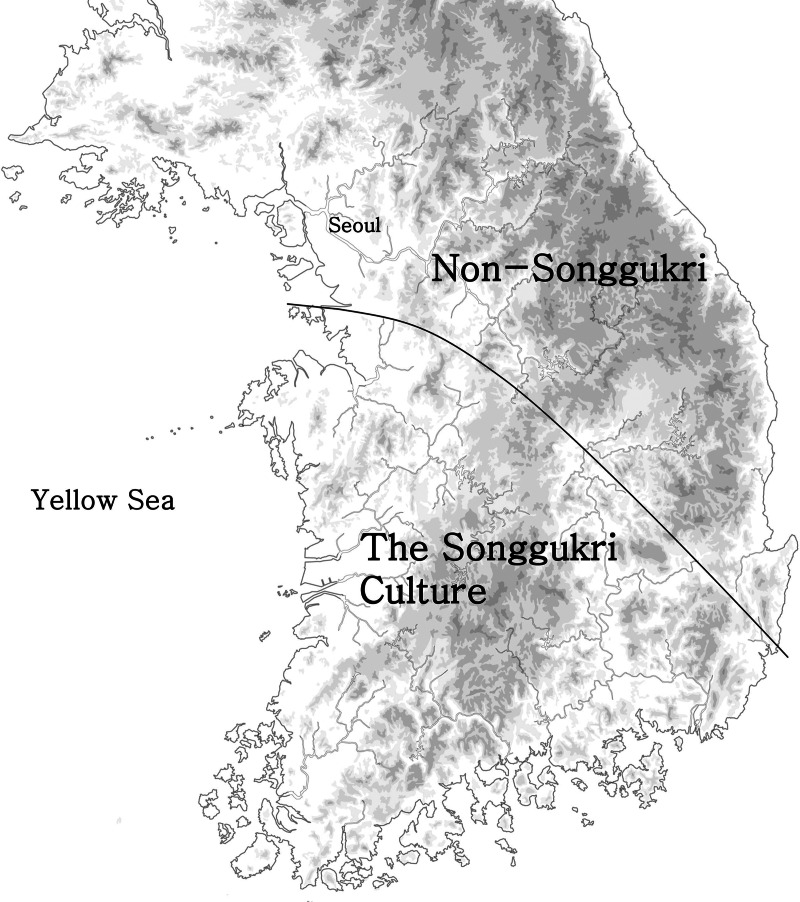
The distribution of the Songgukri Culture and non-Songgukri cultures.

These lines of evidence are in good accordance with many of the criteria for detecting migration listed above (Haury 1958; Rouse 1958; Zvelebil 1981) and suggest that the rapidity of the spread and homogeneity of the new material culture and subsistence economy after their spread throughout the region were brought through the migration of farmers. Spatial analyses of earlier Mumun settlements also confirm that migration was critically involved in the geographic spread of the early Mumun culture in the region (Kim 2002a). The summed probability density of radiocarbon dates from Korea, which archaeologists now widely employ to infer population dynamics, indicates a population boom starting from the beginning of the Mumun Period around 1300 BCE (roughly 3300 calibrated BP, Figure 2) (Oh *et al*. 2017). Nevertheless, the changes in subsistence economy and material culture and population increase should not simply be assumed to be the result of a conquest of farmers or ethnic replacement. Rather, Jangsuk Kim (2002a, 2003a) has suggested that conflicts arising as a result of the different land-use strategies of the two economies (that is, indigenous Chulmun hunter–gatherers’ logistically mobile land use vs migrant farmers’ exclusive territoriality) caused an abrupt increase in the mobility costs of Chulmun hunter–gatherers, straining their subsistence economy. Under these conditions, Chulmun hunter–gatherers probably abandoned their subsistence strategies and were rapidly integrated into the Mumun farming economy. Then, the migration of rice farmers around 1300 BCE seems more likely to have resulted in language dispersal from northeast China to Korea than the introduction of millet in the fourth millennium BCE.

Around 800 BCE, a new material culture complex known as the Songgukri Culture appears in the Geum River Valley and spreads to southwest Korea (Ahn 2010; Grier and Kim 2012; Kim 2003b, 2014), while in central Korea the Early Mumum material culture persists with little change, resulting in the appearance of a spatial division in material culture and subsistence practices (Figure 2). During the ensuing period (800–400 BCE, the Middle Mumun), farming rapidly intensified with the adoption of wet rice farming and there is clear evidence of significantly increased social complexity (Kim 2003b, 2014). While some researchers (e.g. Lee 2005) have attempted to locate the origins of the Songgukri Culture and wet farming outside of the Korean Peninsula, most Korean archaeologists agree that the development of the Songgukri Culture and the spread of wet farming in southwestern Korea were subsistence-technological innovations developed by local populations in order to solve a population–resource imbalance resulting from rapid population growth and reduced soil fertility owing to long, continuous farming in the Early Mumun Period (Kim 2003b). However, it is still unknown why the Songgukri Culture and wet farming techniques remained confined spatially to southwestern Korea and did not spread to central Korea. In our view, the spatial division of material culture and subsistence strategies between central and southern Korea was largely the result of differences in environmental factors. Whereas marshes and bogs are well distributed in southern Korea, they are rare in the more mountainous central Korea, which would have made the construction and use of rice paddies far more costly in that area.

**Figure 3.**
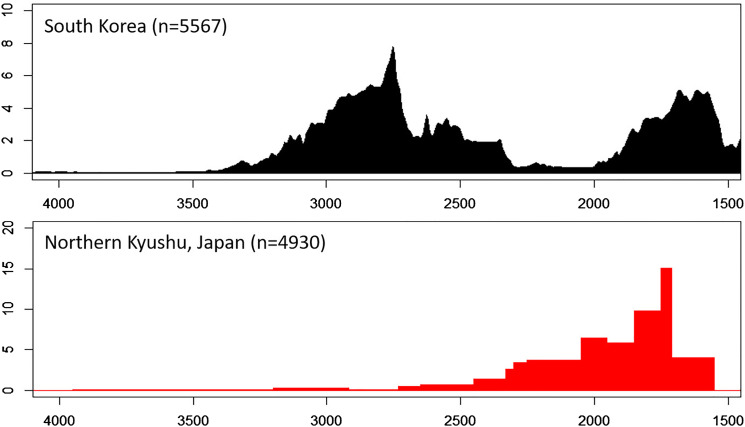
Population fluctuations of South Korea and northern Kyushu, Japan (modified from Oh 2018). Dates on the *x*-axis are in calibrated BP. South Korean population fluctuations are estimated using a summed probability distribution of radiocarbon dates from houses. Population estimations for Northern Kyushu are based on pottery chronology.

It is very likely that around 600–500 BCE part of the Songgukri population of southwestern Korea moved to the Japanese Archipelago, and Korean and Japanese archaeologists agree that wet farming of rice was brought to Kyushu by migrants from southern Korea (Kataoka 1999; Lee 2000; Yoo 2010). Houses, stone tools and ceramics associated with the Songgukri Culture are found as a package in western Japan. Songgukri-style houses, which are very distinctive in shape and structure and thus easily distinguished from local Yayoi houses, have been discovered from 326 settlements out of a total of 710 Yayoi settlements in western Japan, together with Songgukri-style stone tools and pottery (Yoo 2010). Archaeological evidence also suggests that somewhere between 600–400 BCE population decreased on the Korean Peninsula, while Kyushu, Japan witnessed a coincident population increase. While radiocarbon dates indicate that the Japanese Yayoi Period and rice cultivation began as early as 900 BCE, the number of Yayoi sites significantly increased from 600 to 400 BCE, and this increase coincided with the population decline of the Songgukri Culture (Figure 3; Oh 2018). Although the reason for population decline in Korea at this time is not clear, the simultaneity of population decline in Korea and increase in Kyushu, Japan may be best explained by a large-scale migration of Songgukri farmers to Japan.

Around 400–300 BCE, bronze slender daggers and other associated bronze items, such as mirrors and bells, appeared in Korea (Ahn 2010). Since these new bronze items were used earlier in northeast China, some Korean archaeologists have suggested that these were also brought by migrants from northeast China (Ahn 2010; Jo 2005). However, settlements associated with this new material culture are very scarce and sparsely distributed, so it is not clear whether a sizeable migration actually occurred (Kim 2009).

## Evaluating the FLDH in the context of the dispersals and split of the Koreanic and the Japonic languages

As we briefly stated above, the FLDH has recently been applied by historical linguists to infer the timing and routes of dispersals of the proto-Koreanic and proto-Japonic languages (Robbeets 2017b–d; Whitman 2011), with researchers focusing on the spread of millet and rice farming. However, depending on which cultivar is emphasized by linguists, their conclusions diverge significantly. Using archaeological evidence, here we evaluate different arguments on the dispersal and split of the proto-Koreanic and proto-Japonic languages.

### The spread of millet as evidence for language dispersal

Robbeets (2017b–d) pays special attention to the introduction of millet into Korea in the Chulmun Period and attempts to link this process with the appearance of proto-Koreanic in the Korean Peninsula. Relying on the FLDH and also on Gyeong-Ah Lee's argument (Crawford and Lee 2003; Lee 2011) that the introduction of millet eventually culminated in the transition to a farming economy, Robbeets assumes that millet, the first non-local cultivar produced in Korea, was introduced around 3500 BCE by farmers speaking proto-Koreanic migrating from Liaoning in present-day northeast China. However, as outlined above, because the Korean Peninsula was already occupied by Chulmun hunter–fisher–gatherers since at least 6000 BCE, a key to evaluating the millet hypothesis is determining whether millet was adopted by the Chulmun foragers (diffusion) or whether it was brought along as a part of a large-scale migration of farmers from Liaoning. If millet was introduced as a result of a large-scale migration of farmers from Liaoning, an archaeologically detectable influx of Liaoning culture and changes in material culture after the introduction of millet should be expected, because vessel shape, manufacturing technology and the design layout and motifs of Korean Chulmun pottery markedly differ from those of Liaoning pottery. However, there is no detectable appearance of elements of Liaoning material culture that accompanies the arrival of millets. In addition, there is no evidence to suggest that the introduction of millet had a significant impact on the existing Chulmun economy, throwing into question Lee's argument (Crawford and Lee 2003; Lee 2011), on which the millet hypothesis heavily relies. Continuity observed in Chulmun stone tool assemblage strongly suggests that hunting, gathering and fishing persisted as major components of the Chulmun subsistence strategy, and isotope analyses on human bones dating later than 3500 BCE also support the notion that the Chulmun subsistence economy continued to rely heavily on hunting, gathering and fishing (Ahn *et al*. 2015; An 2006; Choy and Richards 2010; Kim 2010). Shortly after the appearance of millet, rather than the aggregation of farming villages, the Chulmun Period witnesses an increase in mobility and dependence on maritime resources and wild plants (Ahn *et al*. 2015; Oh *et al*. 2017). If there had been a large-scale migration of farmers that was substantial enough to lead to linguistic change, we might expect that populations would increase following the migration. However, radiocarbon dates suggest that, while the Chulmun population increased before the appearance of millet, shortly after the introduction of millet around 3500 BCE, the population declined (Figure 1). An alternative explanation for these population trends is that, facing a population increase, Chulmun hunter–gatherers might have indigenously adopted millet as a supplementary resource, but it did not play a significant role in solving the resource–population imbalance. Even if millet was brought by some migrants from northeast China to Korea, archaeological evidence demonstrates that the scale of migration was probably not large enough to lead to a fundamental linguistic change or the dispersal of a linguistic family.

As far as proto-Koreanic language is concerned, the millet hypothesis does not conform well to archaeological records. More importantly, the hypothesis is based on an assumption that the FLDH is a general model, and thus, following the expectations of the FLDH, the first appearance of non-local cultivars in an area determines the timing, cause and process of language dispersal. While some terms associated with millet cultivation might have been introduced along with millet, the available archaeological evidence does not seem to correspond with a large-scale migration that would result in the spread of the proto-Koreanic language.

### Rice and the dispersal of Japonic and Koreanic

In explaining the formation of Koreanic, Whitman (2011) relies less on the FLDH than Robbeets, but still emphasizes the spread of farming to Korea and Japan as the primary mechanism for the dispersion of languages. From an archaeological perspective, the biggest difference is that he focuses on rice rather than millet. He suggests proto-Japonic entered the Korean Peninsula as rice was introduced around 1300 BC at the beginning of the Korean Mumun Period and then moved to the Japanese Archipelago around 800 BCE.

As discussed above, the transition from the Chulmun Period to the Mumun Period in Korea witnessed dramatic and abrupt changes in every aspect of material culture, subsistence technology and settlement patterns. Many lines of evidence indicate that a large-scale migration of farmers and exclusive occupation of resource patches that had been widely exploited by Chulmun hunter–gatherer–fishers led to the collapse of the Chulmun economy and an integration of the indigenous Chulmun groups into Mumun farming societies (Kim 2002a, 2003a). In this sense, the appearance of dry farming of rice in Korea around 1300 BCE is a much more plausible candidate for the dispersal of a new language to the peninsula than the introduction of millet around 3500 BCE. Whitman (2011) also suggests that the migration of wet rice farmers from southern Korea to Japan resulted in the movement of proto-Japonic to Japan. This argument also conforms with the extensive distribution of Songgukri-style material culture in western Japan (Kataoka 1999; Yoo 2010) and demographic changes in both Korea and Japan in this period (Figure 2). Regardless of the universality of the FLDH, archaeological data associated with the spread of rice farming into Korea and Japan correspond well with material expectations we would predict to observe in the process of a potential language dispersal.

However, Whitman's argument that the Koreanic language arrived from northeast China in Korea along with the introduction of the ‘slender bronze dagger’ (also called the ‘Korean-style Bronze dagger’) around 300 BCE deserves further scrutiny. Although some Korean archaeologists (e.g. Jo 2005) argue that the appearance of slender bronze daggers was triggered by the Chinese Yan's attack on Gojoseon, a polity that was located in present-day northeast China, and a subsequent large-scale migration of refugees from Gojoseon to the Korean Peninsula, the nature and size of this migration is unclear. As stated above, the Songgukri Culture witnessed some degree of population decline, but it still densely occupied southern Korea when the slender dagger was introduced. In contrast, settlements associated with the Slender Dagger Culture are extremely sparse and found only in marginal areas, and bronze items associated with the Slender Dagger Culture appear only in elite burials (Kim 2009). Some archaeologists have asserted that the scarcity of Slender Dagger Culture settlements is the result of a shift towards nomadic lifeways (Ahn 2010), but there is no archaeological evidence that suggests that the Slender Dagger people were nomadic.

Alternatively, Jangsuk Kim (2009) has suggested that while there might have been migrants from Gojoseon, they did not dominate the local Songgukri population. Rather, the scarcity of Slender Dagger settlements and their distribution in marginal areas may suggest that elites of the Songgukri Culture selectively accepted a relatively small number of bronze specialists from Gojoseon. If this were the case, the size and sociopolitical power of migrants from Gojoseon would probably not have been large enough to change the local language. This scenario does not lend support to Whitman's suggestion (2011) that the proto-Koreanic language, which apparently differed from the existing Songgukri language (presumably proto-Japonic), entered Korea along with the slender dagger. At present, any archaeological arguments on this issue, especially regarding the size of the migrant population and post-migration relations between migrants (slender dagger producers) and hosts (the Songgukri population), inevitably rely on indirect, circumstantial evidence. Therefore, whether or not the appearance of the slender dagger led to the formation of proto-Koreanic in Korea is not a question that can be addressed with any certainty.

### Some thoughts on proto-Koreanic and proto-Japanic

What does the above archaeological consideration tell us about the spread and divergence of the proto-Koreanic and proto-Japanic languages? Although prehistoric archaeology can provide valuable insight to historical linguistics, archaeologists will tend to remain agnostic on this issue. As many archaeological, anthropological and historical studies have indicated, there is no straightforward connection between material culture, ethnicity, subsistence economy and language (Barth 1969; Binford 1973; Gosden 1999; Jones 1997; Roux 2016), and thus, inferring the formations, expansions and divergences of languages in prehistory directly from the archaeological record requires a great degree of speculation. Despite the potential risks, we here suggest a few hypotheses that must be carefully assessed with further problem-oriented investigations and tests. The first hypothesis is that proto-Japonic and proto-Koreanic had already split (assuming they are genetically related) before they entered the Korean Peninsula. Both proto-Japonic and proto-Koreanic speakers migrated together to the Korean Peninsula along with the dry farming of rice and formed the Mumun Culture. Later, speakers of proto-Japonic aggregated in southern Korea and developed wet farming as an internal innovation in the southern part of the peninsula (i.e. the Songgukri Culture), and later, migrated to Kyushu, Japan.

The second scenario assumes that before the divergence of proto-Japonic and proto-Koreanic, speakers of the common ancestors of the two languages entered the Korean Peninsula, bringing with them dry farming and rice. The Songgukri Culture newly appeared in southwestern Korea ca. 800 BCE, while populations in central Korea preserved the existing material culture and subsistence economy, resulting in a spatial division that eventually led to the divergence of proto-Japonic and proto-Koreanic. In other words, the language spoken by the southern group developed into proto-Japonic, while the language spoken by the remaining central and northern groups became proto-Koreanic. Around 600 BCE, some of the Songgukri population, the speakers of proto-Japonic, migrated to Kyushu, Japan, bringing with them the proto-Japonic language.

Both scenarios have problems. The first scenario assumes a split between proto-Koreanic and proto-Japonic before their entrance into the Korean Peninsula, but as seen above, Early Mumun material culture and technology were homogenous throughout the peninsula, making it difficult to distinguish the two groups. Meanwhile, the second scenario, which assumes the split between the two languages took place as the Songgukri Culture newly appeared in southern Korea around 800 BCE, does not well explain the linguistic distance between Koreanic and Japonic, which is too great to assume a recent split. In short, while the first scenario is not well supported by the archaeological record, the second is not strong enough from a linguistic perspective.

If interpretations are not restricted by the main premises of the FLDH, more hypotheses about the spread and split of proto-Koreanic and proto-Japonic can be proposed. It is now well known that, before the introduction of rice to Korea, hunter–gatherers of the Korean Chulmun, Japanese Jomon and the Boisman Culture of the Russian Far East maintained regional-scale networks from the seventh to third millennia BCE (Korean Archaeological Society 2010). Also, after the end of the Slender Dagger Period around 100 BCE, Chinese Han commanderies were established in northern Korea and iron was widespread and actively used as agricultural tools and weaponry, facilitating the emergence of various competitive polities, spatial divergence in material culture, a total reorganization of international networks and maybe population distributions (Korean Archaeological Society 2010). We do not argue that these events provide a better hypothesis to explain the spread and divergence of the Koreanic and Japonic languages. Instead, we suggest that there are many alternative factors that may possibly explain the dispersal and divergence of the two languages that do not necessarily rely on the FLDH.

## Conclusion

It is not surprising that linguistics is concerned primarily with prehistoric migrations potentially associated with language dispersals when it uses research from prehistoric archaeology and genetic studies. However, before applying archaeological evidence to answering linguistic questions, it is necessary to better understand the structure and processes of migrations of the past. Archaeology has long wrestled with epistemological and methodological issues regarding migration, such as how we detect migration from the archaeological record, how and why people migrated, how migrants adapted to new natural and sociopolitical environments and how we distinguish migration from other mechanisms of spread. Archaeology has realized that distinguishing migration from other mechanisms is an extremely difficult task even when genetic studies are applied. Furthermore, archaeologists have become cognizant that migration is a very complicated process that involves various economic, political, social and environmental factors rather than a simple and singular event or conquest. The spread of subsistence economies, cultivars, technology, ideas, information, material culture and languages should be treated distinctly, and the spread of one cannot be simply linked to the spread of another.

The FLDH is a region-specific hypothesis, not a general model. Although many occurrences of the spread of farming may have been related to migrations of farmers, researchers should use caution when applying the FLDH. Although the spread of language may result from migration, migration does not always lead to language dispersal. Setting aside the question of the FLDH's validity for explaining the Neolithization of Europe, its application to other areas requires a great deal of circumspection. Even if this hypothesis were expanded and renamed the ‘subsistence/demography hypothesis’ (Robbeets 2017a: 19), the same major issues still remain. According to Kristiansen (2005: 680), the FLDH ‘introduced an unhappy marriage between language, archaeology and genetic studies whose ingredients can very easily be misused’.

Particularly in East Asia, drawing links between migration and language dispersals requires great caution. Long-standing public/national interest in the origins of ethnic groups combined with the hyper-migrationist, culture–historical paradigm that has dominated East Asian archaeology have led to an inclination to regard the appearance of new cultural elements as evidence of the migration of distinct ethnic groups. Most, if not all, cultural changes in East Asia have been exaggeratedly interpreted as resulting from the migrations or advent of people with superior technology from other areas (Choi *et al*. 2017). Whitman's and Robbeets’ contrasting understandings of the formation and split of proto-Koreanic and proto-Japonic are probably not only the result of methodological differences. The main contrast between the two is which crop they pick (millet for Robbeets, and rice and the slender dagger for Whitman) as a candidate for the correlate of language dispersal and which archaeological works they rely on (Lee for Robbeets and Ahn for Whitman) to connect changes in material culture to proposed migrations.

No doubt, in order to understand language dispersal, an issue not only of great academic interest but also public interest as well, an interdisciplinary approach is imperative. Nevertheless, this approach should be firmly grounded on thorough understandings of all disciplines contributing to the research.

## Research transparency and reproducibility

Radiocarbon dates for Figure 1 can be found at https://doi.org/10.1017/RDC.2017.122.
